# Cardiac glycosides inhibit early and late vaccinia virus protein expression

**DOI:** 10.1099/jgv.0.001971

**Published:** 2024-03-28

**Authors:** Jerzy Samolej, Ian J. White, Blair L. Strang, Jason Mercer

**Affiliations:** 1 Insititute of Microbiology and Infection, University of Birmingham, Birmingham, UK; 2 Laboratory for Molecular Cell Biology, University College London, London, UK; 3 Institute for Infection and Immunity, St George's, University of London, London, UK

**Keywords:** drug, compound, viral transcription, virion assembly, infection, poxvirus

## Abstract

Cardiac glycosides (CGs) are natural steroid glycosides, which act as inhibitors of the cellular sodium-potassium ATPase pump. Although traditionally considered toxic to human cells, CGs are widely used as drugs for the treatment of cardiovascular-related medical conditions. More recently, CGs have been explored as potential anti-viral drugs and inhibit replication of a range of RNA and DNA viruses. Previously, a compound screen identified CGs that inhibited vaccinia virus (VACV) infection. However, no further investigation of the inhibitory potential of these compounds was performed, nor was there investigation of the stage(s) of the poxvirus lifecycle they impacted. Here, we investigated the anti-poxvirus activity of a broad panel of CGs. We found that all CGs tested were potent inhibitors of VACV replication. Our virological experiments showed that CGs did not impact virus infectivity, binding, or entry. Rather, experiments using recombinant viruses expressing reporter proteins controlled by VACV promoters and arabinoside release assays demonstrated that CGs inhibited early and late VACV protein expression at different concentrations. Lack of virus assembly in the presence of CGs was confirmed using electron microscopy. Thus, we expand our understanding of compounds with anti-poxvirus activity and highlight a yet unrecognized mechanism by which poxvirus replication can be inhibited.

## Introduction

The eradication of smallpox and subsequent discontinuation of smallpox vaccination has left much of the human population susceptible to poxvirus infection. While existing smallpox vaccines (Imvanex and ACAM2000) provide protection against different poxviruses, including mpox (monkeypox), the 2022 mpox outbreak was a reminder of the pandemic potential of these viruses [1,2]. In human populations lacking anti-viral immunity anti-poxvirus drugs are required to treat disease.

There are only two drugs approved for human treatment of smallpox: Tembexa (brincidofovir) approved in the United States and TPOXX (Tecovirimat or ST-246) approved in the United States, United Kingdom and European Union [3,6]. Tembexa is a nucleotide analogue, while TPOXX inhibits F13, a poxviral protein required for production of extracellular virions. Despite their efficacy against diverse human and non-human poxviruses [7], both compounds target viral factors and viruses resistant to these drugs can emerge. This has been observed for both Tembexa and TPOXX *in vitro* [6,8] and for TPOXX *in vivo* [9,10]. Therefore, the identification of additional anti-poxvirus drugs is essential.

Cardiac glycosides (CGs) are a class of compounds historically used to treat cardiovascular conditions, including congestive heart failure and angina pectoris [11]. CGs are natural steroid glycosides composed of a steroid ring, a lactone ring, and a sugar moiety. Additionally, these structures have a variable ring with either five or six carbons, that allows classification of CGs as either cardenolides or bufadienolides, respectively [12]. Some well-known CGs include the cardenolides digoxin and digitoxin derived from *Digitalis purpurea* (foxglove) plants, ouabain from *Strophanthus gratus*, and bufalin, a bufadienolide derived from *Rhinella marina* (cane toad) venom.

Mechanistically, CGs inhibit the cellular sodium-potassium ATPase pump (Na+/K+ATPase) by binding to the extracellular side of these transmembrane enzymes [13,15]. CG binding blocks Na+/K+ATPase activity thereby directly affecting the Na+ and K+ ion concentrations within cells. Inhibition of Na+/K+ATPase by CGs triggers multiple intracellular signalling pathways through activation of kinases, including protein kinase C, protein kinase A, phosphoinositide 3-kinase, protein kinase B and Src tyrosine kinase [16,20]. Therefore, inhibition of Na+/K+ATPase can have pleiotropic effects.

In addition to their role in the treatment of cardiovascular diseases, CGs have also been explored as potential anti-cancer and anti-viral drugs [21,22]. However, due to unwanted off-target effects including cardiovascular complications, cell cycle arrest, the induction of apoptosis and cell death, there has been understandable reticence to use these compounds as anti-viral drugs [23]. Nonetheless, CGs have been shown to inhibit a very wide range of RNA and DNA viruses, including influenza, human cytomegalovirus, herpes simplex virus, coronaviruses and Ebola virus (Table 1). However, the interaction of CGs with poxviruses had not been extensively explored.

**Table 1.** Anti-viral activity of cardiac glycosides (CGs)

Compound	Virus inhibited	References
**Bufalin**(**bufadienolide**)	DENV, HBV, HIV, MERS-CoV, SARS-CoV-2	[48,52]
**Cinobufagin**(**bufadienolide**)	IAV, MERS-CoV, SARS-CoV-2	[42,49]
**Convallatoxin**	HBV, HIV, HCMV	[43,50, 51, 53]
**Digitoxigenin**	HSV, SARS-CoV-2	[46,52]
**Digitoxin**	HSV, DENV, HIV, HCMV, MERS-CoV, SARS-CoV-2	[46,48,52, 54]
**Digoxigenin**	HSV, HIV	[46,50]
**Digoxin**	HSV, VZV, HCMV, AdV, DENV, VACV, HIV, HCMV, CHIKV, ZIKV, EBOV, IAV, SARS-CoV-2	[24,42, 46, 48, 50,52, 55]
**Gitoxin**		[50]
**Lanatoside C**	DENV, VACV, IAV	[24,48, 60]
**Neriifolin**		[24]
**Ouabain**	HSV, HIV, VACV, HCMV, EBOV, IAV, SeV, SARS-CoV-2	[24,43, 46, 50, 52, 54,56, 60, 64, 65]
**Sarmentogenin**		[50]

CGs identified in the literature and viruses they have an effect on. All compounds in the table are cardenolides, except for bufalin and cinobufagin, which are bufadienolides.

See Menger *et al*. [21] and Reddy *et al*. [22] for reviews of anti-viral and anti-cancer activities of CGs, respectively.

AdV adenovirus CHIKV chikungunya virus DENV dengue virus EBOV Ebola virus HBV hepatitis B virus HCMV human cytomegalovirus HIV human immunodeficiency virus HSV herpes simplex virus IAV influenza A virus MERS-CoV Middle East respiratory syndrome coronavirus SARS-CoV-2 severe acute respiratory syndrome coronavirus 2 SeV Sendai virus VACV vaccinia virus VZV varicella zoster virus ZIKV Zika virus

Employing a screen of 2880 chemically diverse compounds for antipoxvirus activity, Deng and colleagues identified cymarine, digoxin, lanatoside C, neriifolin, and ouabain as CGs capable of inhibiting infection or replication of the prototypic poxvirus, vaccinia virus (VACV) [24]. Initial work by Deng and co-workers suggested that these CGs could inhibit vaccinia virus replication at low or sub-micromolar concentrations and were likely to inhibit late poxvirus protein production [24]. However, the mechanistic basis of inhibition has yet to be clarified. These findings prompted us to ask if other CGs could inhibit poxvirus infection and to determine at what stage of the virus lifecycle they act. To this end we applied virological assays to investigate the anti-poxvirus activity of 12 cardenolides and bufadienolides with known anti-viral activities, including four of the compounds discussed above. Overall, we found that all CGs tested had anti-viral activity and were concentration-dependent inhibitors of VACV gene expression.

## Methods

### Cells, viruses, and drug/compounds

HeLa, BSC-40, A-RPE-19 (kind gift from E. Frickel lab, University of Birmingham) were cultured at 37.0 °C/5.0 % CO2 in complete media [Dulbecco’s modified Eagle’s medium (DMEM; Gibco, Life Technologies)] containing 10 % FBS (Sigma) and 1 % penicillin-streptomycin (Pen-Strep; Sigma).

Vaccinia virus (VACV) strain Western Reserve (WR) was used throughout. WT, E-EGFP, l-EGFP [25] and EGFP-A5 [26] have been described. VACVs used were either wild-type (WT) or contained EGFP expressed from the non-essential TK locus under the control of either the VACV J2R early promoter (VACV E-EGFP) or the VACV F17R late promoter (VACV l-EGFP). A5-tagged EGFP was inserted into the endogenous locus to create VACV EGFP-A5. In all cases, mature virions (MVs) were purified from BSC40 cytoplasmic lysates by pelleting through a 36 % sucrose cushion for 90 min at 18 000 ***g***. The pellet was resuspended in 1 mM Tris (pH 9.0). The titre (p.f.u. ml^–1^) was determined by titration on BSC40 cells.

Cycloheximide (CHX; Sigma), cytarabine (AraC, Sigma), and Tpoxx (generously provided by Denis Hruby, SIGA Technologies) were diluted in DMSO and used as indicated throughout the manuscript. Cardiac glycosides (CGs, Prestwick Chemical Libraries) were dissolved in DMSO and used at the indicated concentrations. DMSO was used as a drug carrier control at the same volume as drug or compound used. Cymarine, one of the CGs identified by Deng *et al.* [24] to inhibit VACV replication, was not commercially available to us and, therefore, not assessed in this study.

### Flow cytometry

Experiments were conducted as previously described [27]. Briefly, HeLa cells in 96-well plates were infected with VACV l-EGFP (m.o.i.=0.1) for 24 h (Spread Assay); or VACV E-EGFP l-mCherry MOI=20 for 10 h (EGE and LGE assay). For AraC release experiments, virus was diluted in DMEM containing 10 uM AraC. After 30 min at room temperature the inoculant was replaced with DMEM containing the indicated compounds at the indicated concentrations. For EGE or LGE, cells were infected with VACV the presence CGs at the concentrations indicated in the text and figure legends. After incubation at 37 °C cells were detached with trypsin, followed by addition of 5 % BSA in PBS and fixation with 9 % formaldehyde (FA) in PBS (for a final 3 % FA concentration). The percentage of green-fluorescent cells/all cells was determined using a Guava easyCyte flow cytometer. Gating was done using ‘live cells’ gate first, and then a ‘>99 % of uninfected cells are below threshold’ gate. The results – % of cells expressing EGFP – were then normalised to infected, DMSO-treated controls (DMSO=1). Analysis using GraphPad, [Inhibitor] vs. normalized response -- Variable slope.

### Cytotoxicity

Cytotoxicity was assessed using Abcam’s Quick Cell Proliferation Assay Kit II (WST-1), as per manufacturers’ instructions. HeLa cells (96-well plates) were incubated for 24 h at 37 °C in the various compounds. Concentrations mirrored those used in the flow cytometry Spread Assay experiments. WST solution was added to each well and incubated for 3 h, followed by absorbance measurement at 460 nm, corrected by subtracting absorbance in wells with media but without cells. Values were normalized to cells without any compounds. Data normalized to DMSO control and analysed using GraphPad, [Inhibitor] vs. normalized response – variable slope.

### Virus yield and spread assays

HeLa monolayers (~1.2×10^6^) were infected with VACV WT (m.o.i.=1) in the presence of the specified compounds. For AraC release experiments, virus was diluted in DMEM containing 10 µM AraC before being shifted into the indicated compounds. At 24 h p.i. cells were collected and the pellet resuspended in 100 µl 1 mM Tris (pH 9.0). Cells were freeze-thawed three times to release virus, prior to serial dilution on BSC40 monolayers to determine the p.f.u. ml^–1^ by plaque assay.

### Plaque inhibition assays

A-RPE-19 cells (~5×10^5^) were infected with VACV WT for 48 h in the presence of the specified compound at IC90 (Table 2) at 37 °C. At 48 h p.i. cells were fixed and stained with 0.1 % crystal violet in 4 % formaldehyde (FA). Plate images were digitally captured using a desktop scanner (Cannon).

**Table 2.** CGs IC50, CC50, SI and concentrations used. Half maximal inhibitory concentration (IC50; best-fit-dose response), half maximal cell cytotoxicity concentration (CC50) and selectivity Index (SI) calculated from data shown in Fig. 1. IC90 and IC99 are concentrations used in experiments intended to inhibit infection by at least 90 and 99 %, respectively. All concentrations are displayed in nanomolar

Compound	IC50	CC50	SI	IC90	IC99
**Bufalin**	0.640	65.9	103.0	10	100
**Cinobufagin**	1.24	18.8	15.2	10	100
**Convallotoxin**	0.573	5.72	10.0	5	50
**Digitoxigenin**	17.9	107	6.0	100	500
**Digitoxin**	1.72	167	97.4	10	100
**Digoxigenin**	27.3	247	9.0	100	500
**Digoxin**	4.64	65.9	14.2	10	100
**Gitoxin**	33.9	267	7.9	100	1000
**Lanatoside C**	6.56	134	20.3	100	500
**Neriifolin**	0.344	28.2	82.0	5	50
**Ouabain**	2.88	36.9	12.8	10	100
**Sarmentogenin**	34.8	550	15.8	100	100

### Immunofluorescence microscopy

HeLa cells seeded on CellView slides (Greiner Bio-One) were infected with VACV EGFP-A5 for 30 min at RT. For AraC release experiments, virus was diluted in DMEM containing 10 µM AraC. The inoculant was then replaced with the indicated compounds. After 20 h at 37 °C cells were washed and fixed with 4 % EM grade FA in PBS. Cells were then permeabilized and blocked simultaneously in 0.5 % Triton-X 100/5 % BSA in PBS. Anti-I3 antibody (generously provided by Jakomine Krijnse Locker; Institute Pasteur) was used at 1 : 1000. All secondary antibodies (goat anti-mouse-AF488 and goat anti-rabbit-AF647; Invitrogen) were used at 1 : 1000. Anti-I3 was added for 60 min at RT, followed by a wash and 60 min RT staining with secondary antibody together with DAPI. Images were captured using a 100×oil immersion objective (NA 1.45) on a VT-iSIM microscope (Visitech; Nikon Eclipse TI), using 488 nm and 640 nm laser frequencies for excitation. Images analysed with Fiji [28].

### Virucidal activity assay

VACV l-EGFP (m.o.i=1,000) was pre-incubated with the indicated compounds at IC99 (Table 2) for 60 min at RT in DMEM. The virus mixture was then diluted 1 : 100, resulting in virus equivalent to m.o.i.=10 being added to HeLa cells in complete media. After 30 min of binding at RT the inoculate was replaced with complete media. At 20 h p.i. cells were trypsinised, fixed, and analysed by flow cytometry for EGFP fluorescence.

### Virion binding to cells assay

In total, 50 µl of compounds at 2X IC90 (Table 2) were added to HeLa cells in a 96-well plate on ice. After 15 min, 50 µl of VACV A5-EGFP (m.o.i.=50) was added for 60 min on ice. Cells were then washed with ice-cold PBS, fixed with 4 % FA, and cells analysed by flow cytometry for EGFP fluorescence.

### Electron microscopy

HeLa cells on coverslips were infected at m.o.i. 10 with WT VACV in the presence of AraC for 6 h then shifted into the indicated CGs. At 20 h post-shift, the coverslips were fixed in EM-grade 2 % paraformaldehyde/2 % glutaraldehyde (TAAB Laboratories Equipment) in 0.1 M sodium cacodylate, secondarily fixed for 1 h in 1 % osmium tetraoxide/1.5 % potassium ferricyanide at 4^o^ C and then treated with 1 % tannic acid in 0.1 M sodium cacodylate for 45 min at room temperature. Samples were then dehydrated in sequentially increasing concentration of ethanol solutions and embedded in Epon resin. Coverslips were inverted onto prepolymerised Epon stubs and polymerized by baking at 60^o^C overnight. The 70 nm thin sections were cut with a Diatome 45 diamond knife using an ultramicrotome (UC7; Leica). Sections were collected on 1×2 mm formvar-coated slot grids and stained with Reynolds lead citrate. All samples were imaged using a transmission electron microscope (Tecnai T12; FEI) equipped with a charge-coupled device camera (SIS Morada; Olympus).

## Results

### CGs inhibit VACV cell-to-cell spread

Previously, it was reported that a subset of five CGs (cymarine, digoxin, lanatoside C, neriifolin and ouabain) inhibited VACV infection [24]. To assess if CGs represent a broad class of compounds with anti-poxvirus activity we selected 12 CGs that have been shown to inhibit a wide range of viruses (Table 1). These include ten cardenolides, four of which were previously shown to inhibit VACV (digoxin, lanatoside C, neriifolin, and ouabain [24]), and two bufadienolides, which have not been assessed for anti-poxvirus activity.

**Fig. 1.**
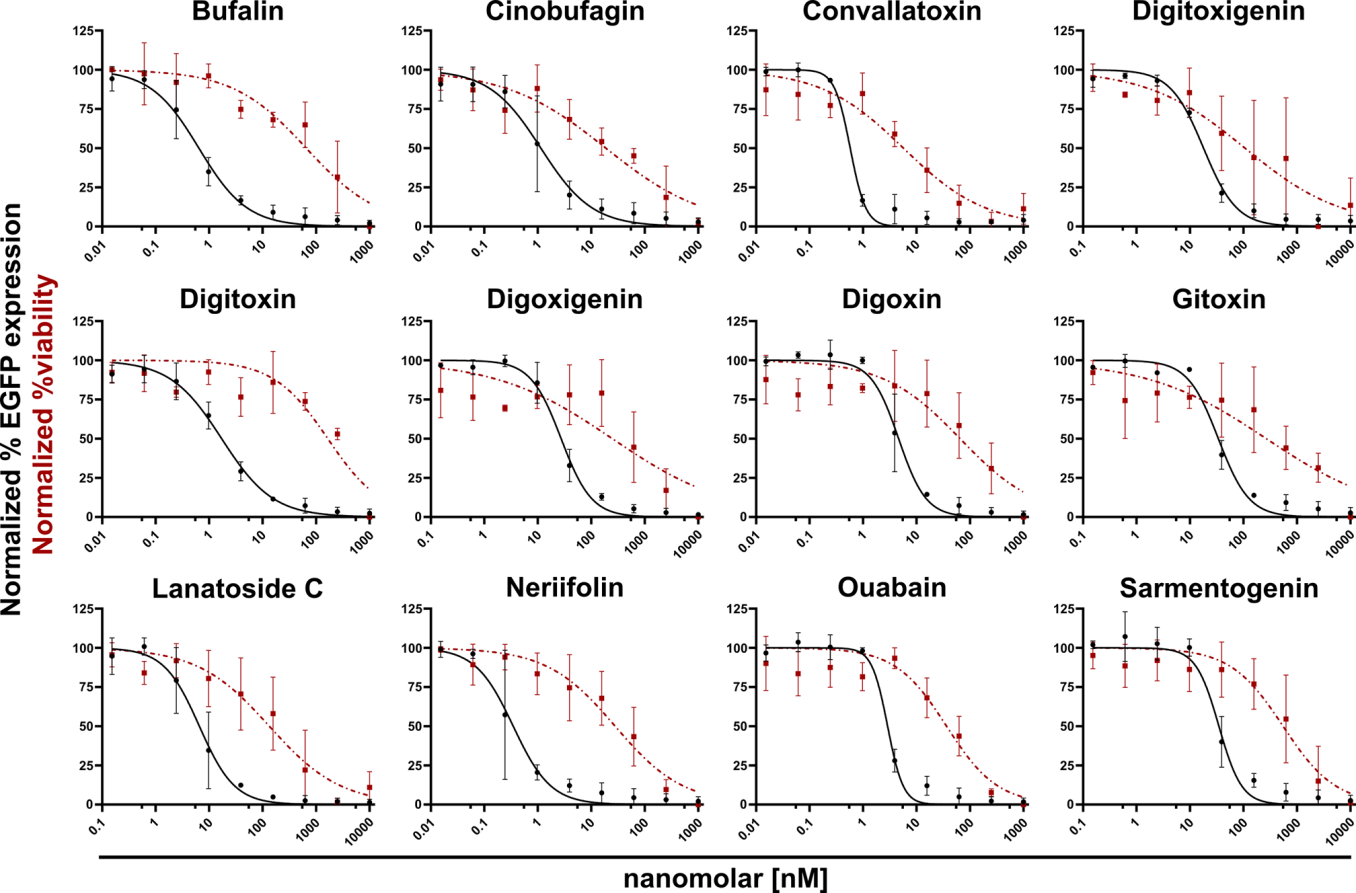
CGs block cell-to-cell spread of vaccinia virus. HeLa cells were infected with VACV l-EGFP (moi=0.1), in the presence of the indicated compounds at various concentrations. 24 h post-infection cells were analysed for EGFP fluorescence by flow cytometry. Percentage of cells expressing EGFP above threshold level displayed as normalised to infected +DMSO=1 (black lines). In parallel, uninfected cells were incubated for 24 h at the same compound concentrations and analysed for cell toxicity using a WST-1 assay. Toxicity is displayed as normalised to infected +DMSO=1 (red dashed lines). Data represents biological triplicates and error bars=sd. Curves: [Inhibitor] vs. normalized response − variable slope.

As it takes only 8 h for VACV to produce viable progeny and spread it to neighbouring cells, a 24 h assay allowed us to look at three complete VACV replication cycles [29,30]. To assess anti-viral activity, we performed a 24 h cell-to-cell spread assay employing a VACV that expresses EGFP from a late VACV promoter [25]. Briefly, HeLa cells were infected with VACV Late-EGFP (VACV l-EGFP) in the presence of various compound concentrations. At 24 h post-infection (h p.i.) cells were analysed for EGFP fluorescence by flow cytometer (Fig. 1a; black lines). A WST-1 cytotoxicity assay was performed using the same drug concentrations on uninfected cells to assure that the anti-viral effects were not due to cell toxicity (Fig. 1a, red lines). All CGs tested showed dose-dependent inhibition of VACV spread with relatively low toxicity within the range of inhibition, including those previously indicated to have anti-poxvirus activity [24]. Determination of the half maximal inhibitory concentration (IC50) and half maximal cell cytotoxicity concentration (CC50) measurements indicated that bufalin, digitoxin, neriifolin, Lanatoside C and Sarmentogenin display high anti-poxvirus activity, without obvious cellular cytotoxicity (Table 2). While all other CGs were still effective at blocking VACV spread, their higher cytotoxicity resulted in lower selectivity index values (Table 2).

### CGs inhibit VACV plaque formation

**Fig. 2.**
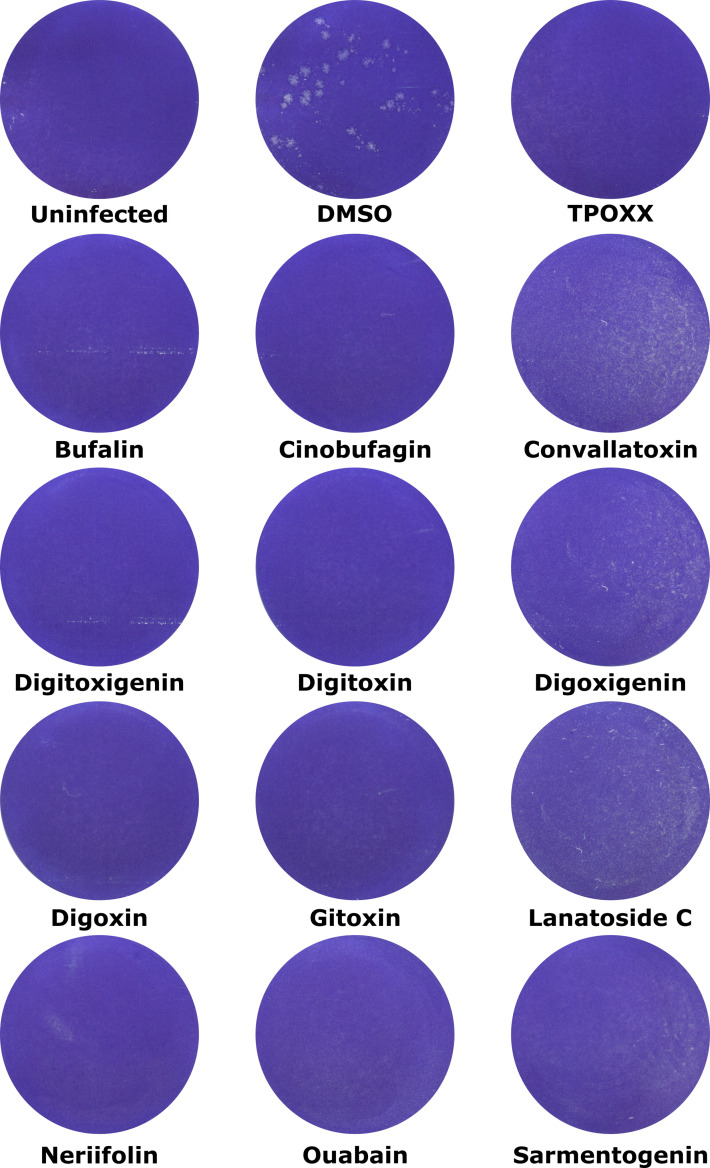
CGs inhibit VACV plaque formation. Monolayers of A-RPE-19 cells were infected with approximately 25 plaque forming units per well of WT VACV in the presence of indicated compounds at IC90. Then, 48 h p.i. cells were fixed and stained with crystal violet and visualized by scanning the plate. Experiments were performed in biological duplicate and representative wells are shown.

To confirm that CGs were effective inhibitors of VACV cell-to-cell spread we performed plaque assays in the presence of the various CGs. As HeLa cells do not form single-cell monolayers due to a lack of contact-mediated inhibition [31], monolayers of A-RPE-19 cells were infected with wild-type (WT) VACV in the presence of DMSO or the CGs. To ensure that the biological effects of the CGs could be readily observed, infections were carried out at concentrations where at least 90 % inhibition of VACV spread could be observed without obvious cellular cytotoxicity in HeLa cell infections (Fig. 1 and Table 2, IC90). Cells infected in the presence of TPOXX, an inhibitor of VACV cell-to-cell spread [6], were also included. At 48 h p.i. cell monolayers were stained to visualize plaques. VACV plaque formation was completely abrogated by all the CGs tested (Fig. 2). Consistent with the calculated CC50 values (Table 2), apart from convallatoxin, digoxigenin and lanatoside C, the cell monolayers appeared largely intact and well-preserved despite 48 h in the presence of the compounds.

### CGs are inhibitors of VACV late protein expression

Cell-to-cell spread assays are used to quantify the passage of infectious virus from one cell to the next, the final stage of the virus replication cycle. Thus, a reduction in virus spread could be due to inhibition of an earlier stage of virus replication ranging from virus binding to the assembly of new infectious virions. As such, we wanted to elucidate what stage of VACV replication was blocked by CGs.

**Fig. 3.**
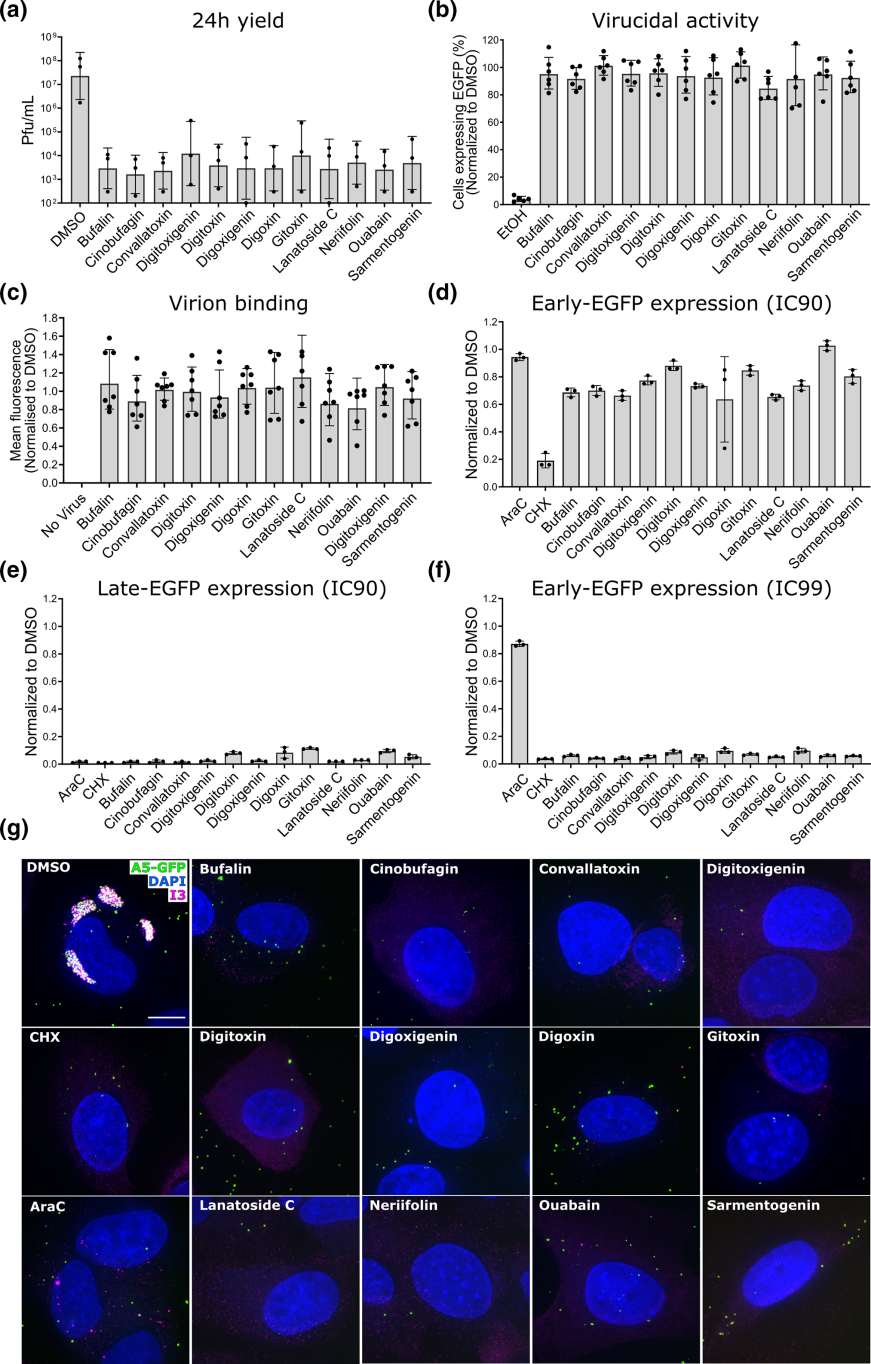
CGs block VACV protein expression and inhibit formation of infectious virions. (a) HeLa cells were infected with VACV WT (moi=1) in the presence of indicated compounds at IC90 (Table 2). Then, 24 h p.i. cells were harvested, lysed, and progeny virus quantified by titration on BSC40 cells. p.f.u. ml^−1^ were normalized to Infected +DMSO=1. Data represents biological triplicates and error bars=sd. Data analysed on GraphPad Prism using ratio paired *t*-test. All compounds significant to at least *P=0.05* compared to DMSO. (b) VACV l-EGFP (EGFP under Late gene promoter) was incubated in indicated compounds at IC99 (Table 2) for 1 h prior to being used to infect HeLa cells. Incubation in 70 % EtOH and DMSO were used as positive and negative controls, respectively. Data represents biological triplicates with technical duplicates and error bars=sd. (c) VACV EGFP-A5 (m.o.i.=50) was bound to cells in the presence of the various CGs at IC99 and analysed for EGFP fluorescence, as a proxy for bound virus. Data normalized to DMSO=1. Data represents biological triplicates with technical duplicates and error bars=sd. (d), (e), and (f) HeLa cells were infected with VACV E-EGFP l-mCherry (EGFP expressed under an early J2R gene promotor, mCherry under a late F17R gene promoter) (d and e) or E-EGFP (EGFP under an early J2R gene promotor) (f) (m.o.i.=10) in the presence of compounds at IC90 or IC99, as indicated. Then, 8 h p.i. cells were analysed for EGFP (green) and mCherry (red) fluorescence by flow cytometry. Values were normalized to infected +DMSO=1. Data represents biological triplicates and error bars=sd. (g) HeLa cells infected with VACV EGFP-A5 (m.o.i.=20) in the presence of indicated compounds at IC99 were fixed 8 h p.i. and immunolabeled for I3. Experiments were performed in biological duplicate and representative images are shown. Scale bar=5 µm.

First, to determine if CGs block the production of infectious progeny virus, we used a 24 h yield assay. HeLa cells were infected with WT VACV in the presence of the various CGs at their IC90s, the concentration that resulted in a 90 % reduction in virus spread (Table 2). At 24 h p.i. cells were harvested and infectious progeny virus quantified. In general, treatment with CGs resulted in a 4-log decrease in 24 h virus yield, apart from digitoxigenin and gitoxin, which lowered virus yield by 2-logs (Fig. 3a). This translates to a 99.99 and 99 % inhibition of infectious virus production, respectively. These results indicated that in the presence of CGs the production of new infectious virions was robustly inhibited.

To understand how virus yield was affected by CGs, we first tested the virucidal activity of the compounds. VACV l-EGFP virions were pre-incubated in the various compounds at their IC99, the concentration that resulted in a 99 % reduction in virus spread (Table 2) for 1 h, washed, and then used to infect HeLa cells. At 8 h p.i. infected cells were quantified and compared to DMSO and 70 % EtOH (negative and positive controls, respectively) (Fig. 3b). Pre-treatment with the various CGs was found to have no obvious impact on virion infectivity, indicating that the compounds had no virucidal effect on VACV particles.

We next assessed inhibition of earlier stages of the virus lifecycle including virion binding and early gene expression (EGE) as well as later stages of the virus lifecycle including late gene expression (LGE). We hypothesized that CGs may have had a profound effect on these steps of VACV replication, as CGs may directly inhibit VACV entry and, therefore, subsequent VACV gene expression. Specifically, CGs inhibit the cellular Na +K+ATPase, which results in significant changes in intracellular ions, increasing Na +and decreasing K+ [32]. These changes have been reported to activate intracellular signalling molecules including those involved in VACV entry, including Src, protein kinase C (PKC) and phosphoinositide 3-kinase (PI3K) [26,33, 34]. In addition, CGs are known to affect the pH of cellular endocytic vacuoles [35,36], such as the macropinosomes used by VACV for internalization.

To test virus binding, a recombinant VACV that encodes an EGFP-tagged core protein A5 found in the VACV virion (VACV EGFP-A5), was bound to cells at an MOI of 50 on ice [26]. Thirty minutes after binding, cells were washed to remove unbound particles and samples analysed by flow cytometry for the mean fluorescence intensity, a proxy for how many virions bound to the cells (Fig. 3c). Relative to DMSO controls, there was no obvious difference in VACV binding to cells in the presence of any of the CGs tested.

To test the impact of CGs on VACV early gene expression (EGE) and late gene expression (LGE) we infected HeLa cells in the presence of the various CGs (IC90 concentration) with a recombinant VACV that expresses both EGFP under the control of a VACV early promoter and mCherry under the control of a late VACV promoter (VACV E-EGFP l-mCherry) [37]. Cells infected in the presence of cycloheximide (CHX), which blocks EGE, or cytarabine (AraC), which blocks viral DNA synthesis and LGE, were included as positive controls. At 8 h p.i. cells were harvested and analysed for green (EGE) and red (LGE) fluorescence by flow cytometry (Fig. 3d, e). While there was variable inhibitory effect on EGE with each CG (Fig. 3d), CGs nearly completely abrogated LGE in infected cells (Fig. 3e).

### CGs inhibit VACV early protein expression at IC99 concentrations

The mechanisms and source of VACV EGE and LGE are different; EGE occurs from viral genomes within VACV cores prior to uncoating and replication of the viral DNA, LGE occurs from free replicated viral genomes in the cytoplasm [38]. This could, in part, explain differences in inhibition of EGE and LGE in the presence of CGs. However, we wished to determine if there were conditions under which CGs could also robustly inhibit EGE, for example, at high concentrations of CGs. Thus, HeLa cells were infected with VACV E-EGFP in the presence of CGs at their respective IC99s: concentrations in which VACV spread is inhibited by 99 % (Table 2). At 8 h p.i. cells were harvested and analysed for green (EGE) fluorescence by flow cytometry. Here, we found that at IC99 CG concentrations EGE was robustly inhibited (Fig. 3f).

To confirm and extend these observations, we infected HeLa cells with VACV expressing EGFP-A5 [a late VACV protein (A5) fused to EGFP that can be visualised in replication sites in the cell, as well as in the cores of mature virions] in the presence of CGs at IC99 concentrations. Cells infected in the presence of cycloheximide (CHX), which blocks EGE, or cytarabine (AraC), which blocks viral DNA synthesis and LGE, were included as positive controls, as were cells infected in the presence of DMSO, which acted as a negative control. At 8 h p.i. cells were immunolabeled for the VACV early dsDNA binding protein, I3, and stained for DNA using Dapi. When analysed by fluorescence microscopy, DMSO-treated cells contained large cytoplasmic VACV replication sites positive for both I3 (magenta) and A5 (green) (Fig. 3g). As expected, cells treated with CHX contained green puncta, consistent with stabilization of EGFP-A5 cores in the absence of EGE [39], and were negative for I3/A5 positive replication sites. Cells treated with AraC contained I3-positive DNA replication initiation sites, which represent viral DNA that was released from virions, but not replicated [39,40]. In all instances, cells infected in the presence of CGs were phenotypically similar to CHX-treated samples. They contained stabilized EGFP-A5 cores and showed no evidence of EGE, LGE or DNA replication initiation site formation (Fig. 3g). This finding was consistent with the ability of CGs to inhibit VACV EGE, and/or LGE, when used at IC99 concentrations (Fig. 3f).

### CGs inhibit VACV late protein expression and reduce infectious virus yield at IC90 concentrations

To better understand the CG block to LGE, we used AraC shift experiments [39] to examine the relationship between blocks to EGE and LGE. As mentioned, VACV gene expression is temporal with EGE occurring before DNA replication and LGE occurring after DNA replication [38]. As such, any drug that blocks VACV EGE indirectly blocks DNA synthesis and subsequent LGE. As we determined that the CGs block EGE at IC99 concentrations, in order to specifically investigate any impact of CGs on LGE, we needed to bypass the CG EGE block. To achieve this, cells are infected in the presence of AraC, which allows for EGE but not DNA replication. AraC is then washed out and infection allowed to proceed in the absence or presence of the CGs at the IC90 concentrations. Thus, infections are shifted from the presence of AraC to CGs. This experimental setup allowed us to assess the impact of IC90 CG concentrations on post-EGE stages of the VACV lifecycle, including DNA replication, LGE and virus assembly.

**Fig. 4.**
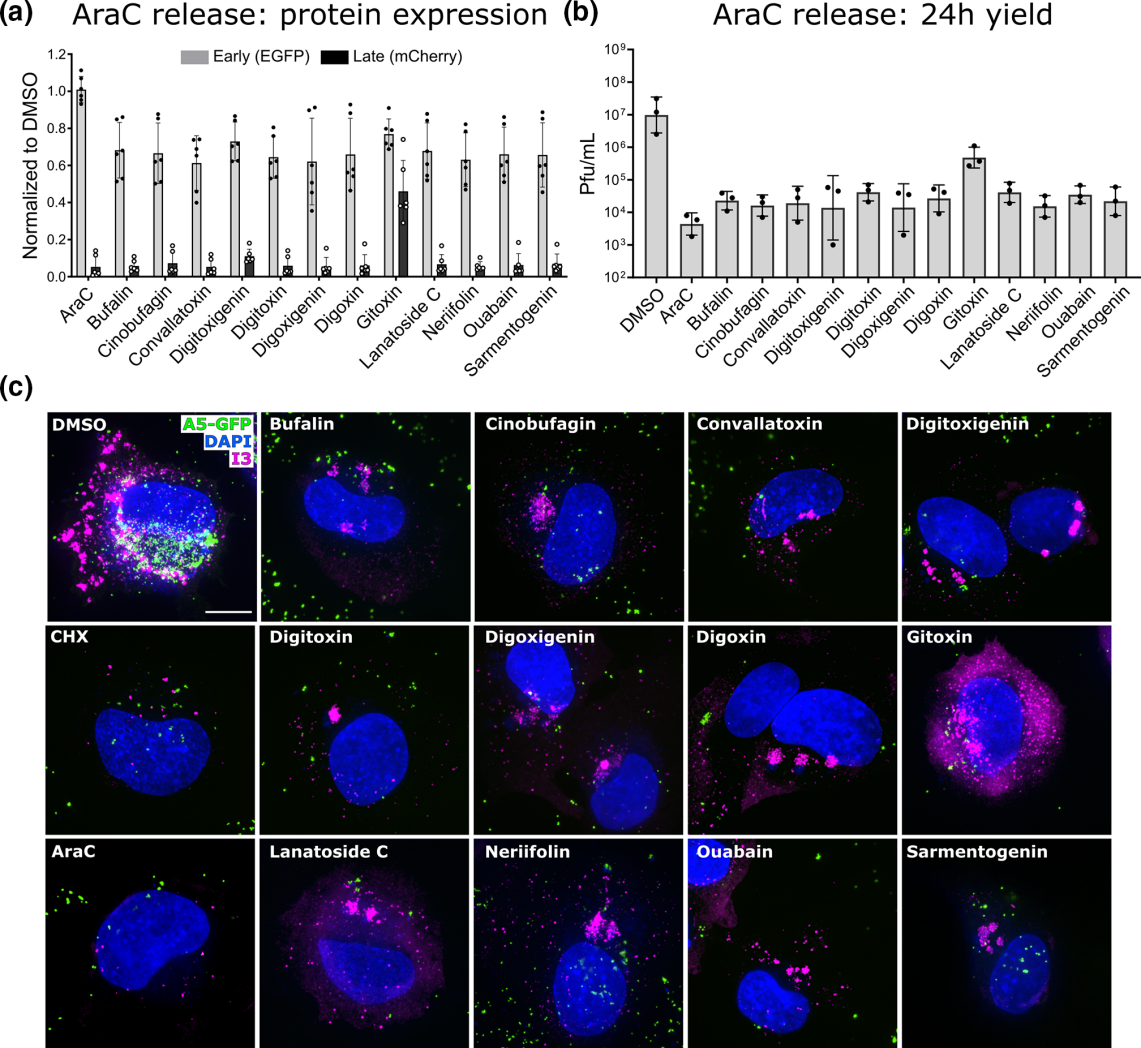
CGs inhibit late protein expression and affect VACV replication site formation. (a) HeLa cells infected with VACV E-EGFP l-mCherry (m.o.i.=10) (EGFP expressed under an early gene J2R promotor, mCherry under a late F17R gene promoter) in the presence of AraC for 6 h then shifted into indicated CGs at IC90. Then, 16 h after CGs addition cells were analysed for GFP and mCherry fluorescence by flow cytometry. Data normalized to infected +DMSO=1. Data represents biological triplicates with technical duplicates and error bars=sd. (b) HeLa cells were infected with VACV WT (m.o.i.=1) in the presence of AraC for 6 h then replaced with indicated CGs at IC90. At 24 h after AraC wash-off and progeny virus quantified. Values normalized to Infected +DMSO=1. Data represents biological triplicates and error bars=sd. Data analysed on GraphPad Prism using ratio paired *t*-test. All compounds significant to at least *P=0.05* compared to DMSO. (c) HeLa cells infected with VACV EGFP-A5 at m.o.i. 20 in the presence of AraC for 6 then shifted into indicated CGs at IC90 for 18 h before immunolabeling for I3. Experiments were performed in biological duplicate and representative images shown. Scale bar=5 µm.

To confirm the AraC-CG shift experiments performed as expected, we assessed VACV EGE and LGE expression. Hela cells were infected with VACV E-EGFP l-mCherry in the presence of AraC for 6 h prior to wash out and subsequent addition of CGs. At 16 h after CG addition, infected cells were analysed for EGE and LGE by flow cytometry. When normalized to an AraC-DMSO shift control, EGE had resumed in all samples after AraC release, indicating that the first CG block had been successfully bypassed (Fig. 4a; grey bars). However, LGE was greatly reduced (>90 %) by all CGs, except for Gitoxin which showed a more modest reduction in LGE (54%) (Fig. 4a; black bars).

Additionally, we performed an AraC-CG shift experiment and measured the 24 h virus yield. Cells were infected with VACV WT in the presence of AraC for 6 h, washed and shifted into CGs. At 24 h post-shift cells were harvested, and the infectious virus yield determined by plaque assay. In most instances a 2.0–2.5 log drop in virus yield was observed (Fig. 4b). Consistent with its impact on LGE, Gitoxin showed the least effect, reducing virus yield by 1 log. These results demonstrate that CGs can block LGE, and subsequent infectious virus production, independent of their impact on EGE.

### CGs compromise replication site formation and block virus assembly

VACV DNA replication is required for VACV LGE. As AraC blocks viral DNA replication, it was possible CGs were acting on either DNA replication or LGE directly. To test this, we performed an AraC-CG shift imaging assay. HeLa cells were infected with VACV EGFP-A5 in the presence of AraC for 6 h and then shifted into CGs at their IC90 concentrations for 18 h. Cells shifted into DMSO, CHX or AraC treated infected cells were included as controls. Cells were immunolabeled for I3 and stained for DNA using Dapi. Cells shifted into DMSO contained large cytoplasmic I3-positive replication sites (magenta) and nascent virions containing EGFP-A5 (green) (Fig. 4c). Cells shifted into with CHX or AraC contained I3-positive DNA replication initiation sites, as expected [39,40]. When cells were shifted from AraC into CGs we observed small, often dispersed, viral replication sites positive for I3 (magenta) (Fig. 4c). Consistent with its lesser impact on LGE and virus yield observed in AraC-CG shift assays (Fig. 4a, b), gitoxin-treated samples were more readily stained for the early protein I3. Consistent with the lack of LGE detected in the presence of CGs (Figs3e  4a), no EGFP-A5 signal (green) was observed within the replication sites of CG-treated samples (Fig. 4c). These results suggested that CGs partially compromise VACV replication compartment development. Of note, despite the formation of VACV replication sites, CGs completely attenuated VACV late protein expression.

**Fig. 5.**
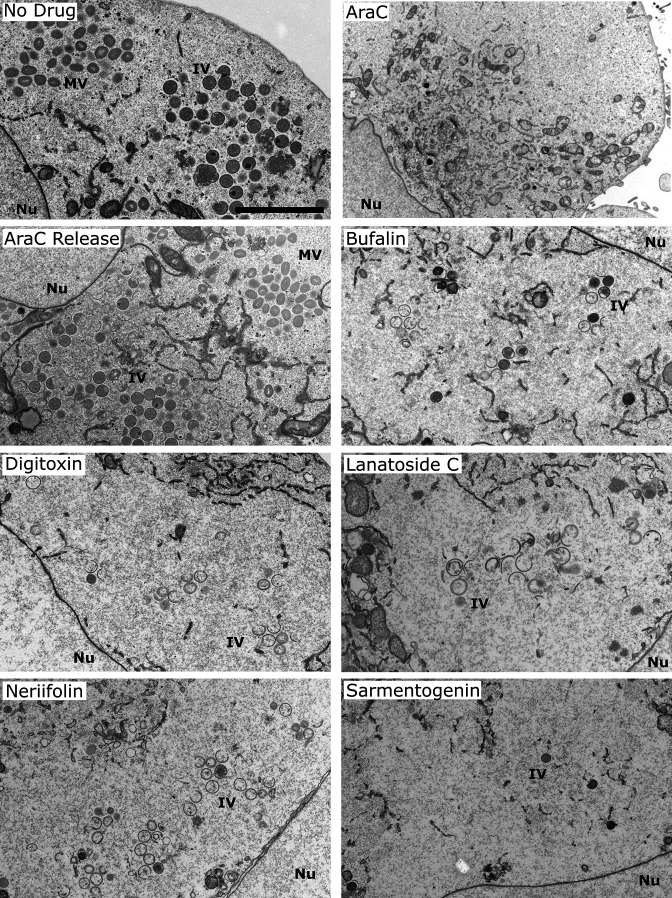
Inhibition of late protein expression by CGs prevents VACV assembly. HeLa cells were infected with WT VACV (m.o.i. 10) in the presence of AraC for 6 h then shifted into the indicated CGs at IC90 for 18 h. Samples were analysed by EM for replication sites and viral assembly intermediates. Experiments were performed in biological triplicate and representative images shown. Scale bar=2 µm.

To better characterize how CG-mediated termination of late protein expression affected VACV production, we investigated the presence of VACV assembly intermediates [crescents or immature virions (IVs)]. As these viral intermediates are not detectable by immunofluorescence [41], we employed electron microscopy (EM) to visualize VACV replication sites. Cells were infected in the presence of AraC for 6 h then washed and shifted into the presence of CGs with the highest calculated SI (bufalin, digitoxin, lanatoside C, neriifolin and sarmentogenin; Table 2) to assist with observing features due to anti-viral effects and not cellular cytotoxicity. Infection was allowed to proceed for a further 18 h prior to fixation and preparation for EM. No drug, AraC and AraC release controls were also analysed. In no drug and AraC release control cells, VACV replication sites containing crescents and immature virions as well as mature virion (MV) clusters were observed (Fig. 5; no drug and AraC release). In AraC-treated control cells, cytoplasmic clearing of organelles was seen with no discernible replication sites or viral structures (Fig. 5; AraC). In all the AraC-CG shift samples, cytoplasmic replication sites devoid of cellular organelles were seen. Confirming and extending the AraC-CG shift imaging experiments (Fig. 4c), the replication sites were always surrounded by discontinuous ER membrane and in most cases contained a small number of viral crescents and IVs with no MVs (Fig. 5). Consistent with the observed reduction in virus yield observed in the presence of the CGs, these results demonstrated that inhibition of LGE by CGs resulted in attenuation of virus assembly at the earliest stages. Collectively, they confirm that CGs can inhibit two independent stages of VACV replication, pre-replicative early protein expression at IC99 and post-replicative late protein expression at IC90 concentrations, respectively.

## Discussion

CGs have been shown to inhibit replication and infectivity of a diverse range of RNA and DNA viruses. Previously, five CGs were found to inhibit poxvirus infection in a large compound screen [24]. Here we characterize the anti-poxvirus activity of a range of CGs and find that they inhibit VACV replication and assembly due to inhibition of early and late VACV protein expression.

CGs are a group of natural compounds primarily used in the treatment of cardiovascular disease, including congestive heart failure and arrhythmias [11]. They inhibit the cellular Na +K+ATPase, which results in significant changes in intracellular ions, increasing Na +and decreasing K+ [32]. The reported effects of these compounds on cellular signalling and endocytic vacuole pH [26,33,36], were likely to have profound effects on the earliest stages of VACV replication. However, we find that VACV virions and the early stages of VACV infection, binding and EGE, are not affected by CGs at concentrations that reduce virus yield by 3–4 logs. At these IC90 concentrations we found that the CGs robustly abrogated VACV LGE. That VACV late genes encode all the virus structural proteins and enzymes required for VACV assembly explains the impact of CGs on virus yield.

CGs have been reported to inhibit a diverse range of viruses, such as human cytomegalovirus (HCMV) and influenza A virus (IAV) by CGs. In HCMV infected cells CGs blocked immediate-early gene expression, whereas in IAV infected cells CGs blocked virus protein translation without affecting virus induced signalling or entry [42,43]. In the case of IAV, the observed defect in viral protein expression correlated with the inhibition of cellular protein translation [42]. The importance of intracellular cationic concentrations on protein translation and the reliance of all viruses on host translational machinery may, in part, explain the broad anti-viral activity of CGs [44].

We also asked if higher concentration CGs (IC99s) could inhibit VACV EGE. We found CGs used at their respective IC99s were effective inhibitors of VACV early protein expression. Taking advantage of this, in combination with an image-based AraC release assay [45] we showed that at these high concentrations, CGs block EGE and subsequent viral genome uncoating, as well as LGE and subsequent virus assembly. Of note, despite the formation of detectible I3-positive replication sites, relative to control samples VACV DNA replication site formation appeared highly compromised in the presence of CGs. EM confirmed that VACV morphogenesis was blocked with only a few viral intermediates present in CG-treated samples.

It is interesting to speculate why differences in VACV protein expression are found using different concentrations of CGs. It is possible that the different sensitivities are due to the fact that EG transcription occurs from genomes within VACV cores, while LG transcription occurs in the cytoplasm from newly replicated VACV genomes. Alternatively, it is possible that the translational fitness of LGs is more sensitive to CGs than EGs. This would be consistent with the finding that early VACV mRNAs are more abundantly expressed than late mRNAs during the course of VACV infection [46].

Cytotoxicity assays indicated that, at least in our experimental models, that CG-mediated VACV inhibition was unlikely due to cytotoxicity. Similar to data presented by Deng and co-workers [24], all of the compounds tested here demonstrated nanomolar IC50 values. This indicates that these compounds are potent inhibitors of VACV replication and have *in vitro* anti-viral activity similar to currently used anti-poxvirus drugs Tembexa and TPOXX [4,6]. Using the IC50 and CC50 values determined in our study to calculate the selectivity index of compounds, the CGs can be broadly classified by their anti-viral potency: high potency: (SI >50; bufalin, digitoxin, and neriifolin), moderate potency: (SI >10; cinobufagin, digoxin, lanatoside C, ouabain, and sarmentogenin) and low potency: (SI ≤10; convallotoxin, digitoxigenin, digoxigenin, and gitoxin). We note that these data differ subtly from that discussed by [24]. For example, Deng and co-workers reported that neriifolin was the least effective inhibitor of poxvirus replication in their experiments, whereas we find it to be one of the most potent. This could be due to minor differences in experimental procedure between our two studies, which suggests that in future studies it will be important to test CG compounds in a number of experimental conditions.

Regarding the therapeutic potential of CGs as poxvirus anti-virals, despite the relatively low toxicity observed at inhibitory concentrations, for most CGs the ‘therapeutic’ window was found to be narrow. Thus, our data supports further testing of more CGs to determine their therapeutic effects and/or the design of CG analogues with improved anti-poxvirus activity.

Regardless of the path forwards for development of CGs as anti-poxvirus agents, there has been understandable reticence to use CGs as anti-viral drugs in systemic applications due to their off-target effects, which may lead to unwanted cardiovascular complications. As poxvirus infection manifests itself as skin lesions, topical application of low concentrations of CGs may be sufficient to treat poxvirus infection [47]. This however, will require further assessment of the anti-poxvirus activity of CGs in relevant skin cell types, due to the unwanted off target effects of CGs such as cell-cycle arrest and induction of apoptosis [23]. Thus, a more thorough understanding of the mechanism(s) by which CGs inhibit poxvirus infection and the development of refined, non-immunosuppressive CGs will need to be considered.

Finally, as we have demonstrated that the CGs studied here have a mechanism of action different from Tembexa and TPOXX, it is possible that CGs could be developed to treat poxviruses resistant to either or both Tembexa and TPOXX.
